# Demographic, clinical, immunological, and molecular features of iranian national cohort of patients with defect in *DCLRE1C* gene

**DOI:** 10.1186/s13223-023-00768-5

**Published:** 2023-02-21

**Authors:** Soodeh Ghadimi, Mahnaz Jamee, Hassan Abolhassani, Nima Parvaneh, Nima Rezaei, Samaneh Delavari, Mahnaz Sadeghi-Shabestari, Sedigheh Rafiei Tabatabaei, Alireza Fahimzad, Shahnaz Armin, Zahra Chavoshzadeh, Samin Sharafian

**Affiliations:** 1 School of Medicine, Azad University of Medical Sciences, Tehran, Iran; 2 Pediatric Nephrology Research Center, Research Institute for Children’s Health, Shahid Beheshti University of Medical Sciences, Tehran, Iran; 3 Immunology and Allergy Department, Mofid Children’s Hospital, Shahid Beheshti University of Medical Sciences, Tehran, Iran; 4 Research Center for Immunodeficiencies, Pediatrics Center of Excellence, Children’s Medical Center, Tehran University of Medical Sciences, Tehran, Iran; 5 Division of Clinical Immunology, Department of Biosciences and Nutrition, Karolinska Institutet, Karolinska University Hospital, Huddinge, Stockholm, Sweden; 6 Department of Immunology and Allergy, Tabriz University of Medical Sciences, Tabriz, Iran; 7 Pediatric Infections Research Center, Research Institute for Children’s Health, Shahid Beheshti University of Medical Sciences, Tehran, Iran

**Keywords:** Inborn errors of immunity, Primary immunodeficiency, Severe combined immunodeficiency, Artemis, DCLRE1C, HSCT

## Abstract

**Background:**

*DCLRE1C* gene mutation leads to Artemis deficiency, a severe form of combined immunodeficiency (SCID). Impaired DNA repair and block in early adaptive immunity maturation results in T-B-NK+ immunodeficiency associated with radiosensitivity. Recurrent infections early in life are the main characteristic of Artemis patients.

**Method:**

Among 5373 registered patients, 9 Iranian patients (33.3% female) with confirmed *DCLRE1C* mutation were identified since 1999–2022. The demographic, clinical, immunological and genetic features were collected through retrospective investigation of medical records and using next generation sequencing.

**Results:**

Seven patients were born in a consanguineous family (77.8%). The median age of onset was 6.0 (5.0–17.0) months. Severe combined immunodeficiency (SCID) was clinically detected at a median (IQR) age of 7.0 (6.0–20.5) months, following a median diagnostic delay of 2.0 (1.0–3.5) months The most typical first presentation was pneumonia (44.4%) and otitis media (3.33%), followed by BCG lymphadenitis (22.2%) and gastroenteritis (11.1%). The most prevalent manifestations were respiratory tract infections (including otitis media) (66.6%) and chronic diarrhea (66.6%). In addition, juvenile idiopathic arthritis (P5) and celiac disease and idiopathic thrombocytopenic purpura (P9) as autoimmune disorders were reported in 2 patients. All patients had reduced B CD19+ and CD4+ cell counts. IgA deficiency occurred in 77.8% of individuals.

**Conclusion:**

Recurrent infections particulary respiratory tract infection and chronic diarrhea during the first months of life in patients born to consanguineous parents should raise the suspicion for inborn errors of immunity, even in the presence of normal growth and development.

**Supplementary Information:**

The online version contains supplementary material available at 10.1186/s13223-023-00768-5.

## Introduction

**Fig. 1:**
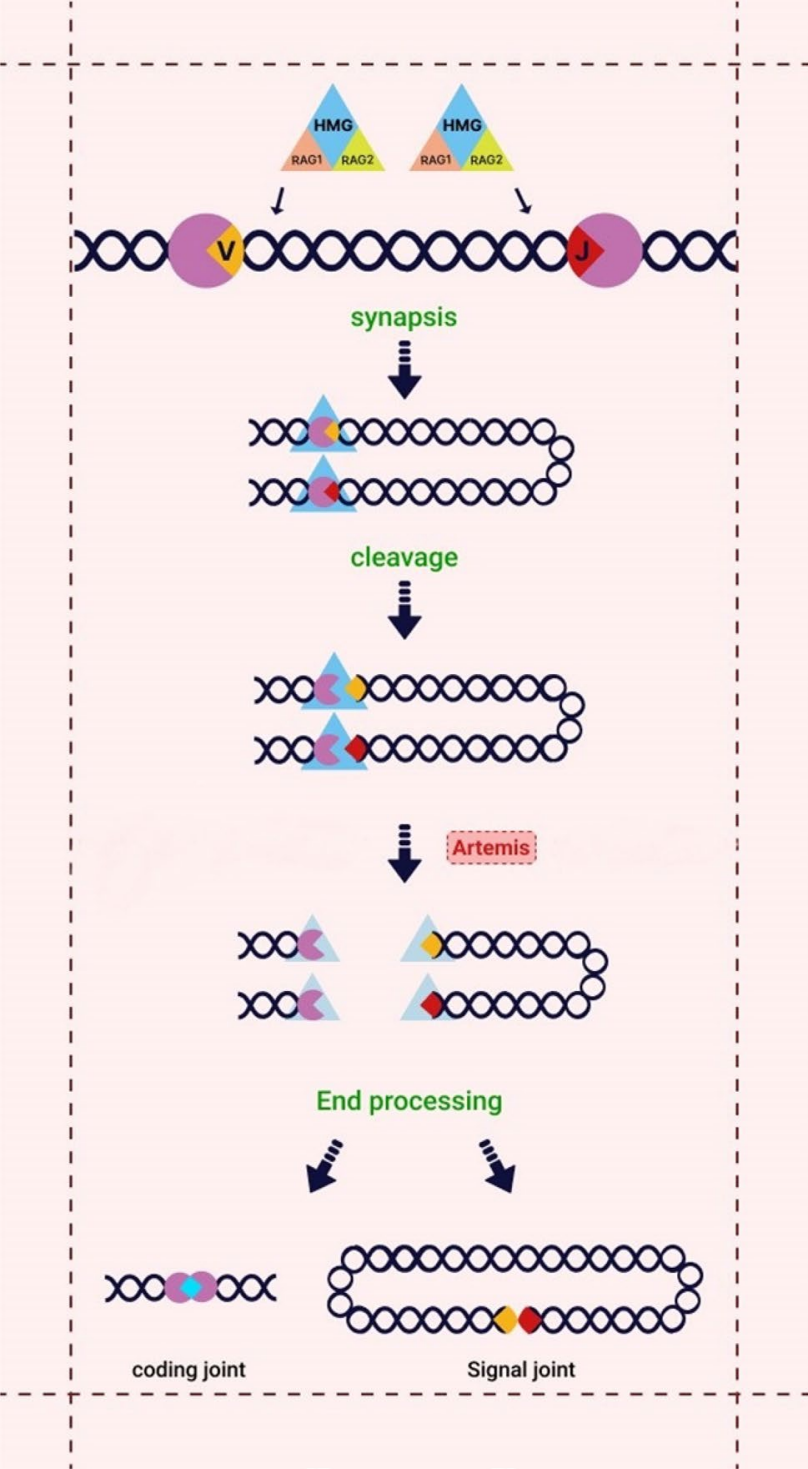
Artemis function in VDJ recombination**.** RAG complexes(RAG1 and RAG2) together with HMG create a synaptic complex by joining two disparate RSS. Upon binding, the RAG proteins generate hairpins at the coding ends and double-strand breaks are introduced. DNA-PK phosphorylates Artemis which opens the hairpins during the non-homologous end-joining pathway of DNA repair

Artemis, an endonuclease protein encoded by *DCLRE1C*, is a crucial component of V(D)J recombination and the non-homologous end-joining (NHEJ) pathway, belonging to the Metallo-β lactamase superfamily [1]. V(D)J recombination known to be a prerequisite for the generation of antigen diversity as well as the early development of T and B lymphocytes, can be divided into three phases: the initial phase mediated by products of recombination activating gene (RAG1) and (RAG2) terminates in the introduction of DNA double-strand break (DSB) and DNA hairpins. During the intermediate phase, in which the Non-homologous end joining (NHEJ) pathway leads to double-strand break (DSB) repair, Artemis plays a key role in opening hairpins at coding ends, which rejoin in the last step [2]. (Fig. 1).

Biallelic DCLRE1C/Artemis loss-of-function impairs the NHEJ pathway and V(D)J recombination which results in the generation of inborn errors of immunity (IEI) mainly with complete penetrance of T-B-NK+ severe combined immunodeficiency (SCID) associated with radiosensitivity [3, 4]. *DCLRE1C* deleterious mutation with high incidence was first detected in Athabascan-speaking Native Americans (SCID-A), mainly presenting recurrent infections and failure to thrive. Other unique features of SCID-A were painful ulcers in the mouth and genital area as well as a lack of growth of secondary teeth [5, 6]. Artemis can present with chronic or recurrent diarrhea, growth failure, and frequent opportunistic and ordinary infections soon after birth which is usually resistant to standard medical treatment [7–9]. In addition, patients with mutations in genes involved in DNA repair are theoretically at increased risk of malignancy [10, 11].

According to registered cases in the Iranian National Registry of Primary Immunodeficiencies (IPIDR), among patients with a definite genetic diagnosis, a mutation in *DCLRE1C* had a prevalence of 0.5%. However, affected patients along with other forms of SCID and non-syndromic CID showed the highest mortality rate (57.2%) [12, 13]. In addition to conventional treatment with prophylactic antibiotics and immunoglobulin (Ig) replacement therapy, hematopoietic stem cell transplantation (HSCT) provides a life-long treatment for children with DCLRE1C defect, similar to other forms of SCID. However, the application of alkylator agents for conditioning regimens is limited due to their toxic effects in radiosenstivie SCID patients [11, 14]. Due to the importance of early diagnosis and prompt treatment of these patients, this study presents a report on demographic, clinical, molecular, and immunological features of Iranian patients cohort with a defect in the *DCLRE1C* gene.

## Method

### Study design and population

This retrospective multi-central study included all recorded patients with DCLRE1C defect, referred to the immunology clinics of Mofid Children’s Hospital, Pediatric Center of Excellence of Tehran University of Medical Sciences, and registered patients in the Iranian primary immunodeficiency registry (IPIDR) with 5373 IEI patients during 1999–2022 [15]. The diagnosis of SCID was made according to the diagnostic criteria of the ESID (European Society for Immunodeficiency) registry working party, later confirmed by the identification of pathogenic variants in the *DCLRE1C* gene using next generation sequencing and as described previously American College of Medical Genetics and Genomics criteria [13]. The study was approved by the ethics committee of Shahid Beheshti University of Medical Science, Tehran, Iran (Approval code: IR.SBMU.RETECH.REC.1401.332), and written informed consent were obtained from parents/legal gurdiann.

### Data acquisition

Informed questionnaires were designed querying the following data through investigating registry/hospital documents and direct interviews by calling intended patients or their parents. These data include: (I) demographics data: age, sex, parental consanguinity, ethnicity, first presentation, age of diagnosis, diagnostic delay, family history; (II) clinical manifestations: autoimmunity, infection, allergic diseases, lymphoproliferative disorders and malignancies, etc.; and (III) laboratory data: complete blood count, lymphocyte subsets, and serum immunoglobulins levels (IgM, IgG, IgA, IgE), lymphocyte transformation test (LTT), all therapeutic agents received including intravenous Ig (IVIg), immunosuppressive, antimicrobials, and hematopoietic stem cell transplantation. Following clinical diagnosis based on the European Society for Immunodeficiencies (ESID) criteria and International Union of Immunological Societies (IUIS) classification, blood samples from patients were analyzed through Whole Exome Sequencing and the pathogenicity of identified genetic variants were scored according to American College of Medical Genetics (ACMG) guideline. The diagnosis was confirmed by Sanger sequencing, and the results were shared with parents for decision making and prenatal counseling for future pregnancies.

### Statistical analysis

Statistical analysis was performed using the SPSS software package (SPSS Statistics version 26.0, Chicago, Illinois, USA). For quantitative variables with abnormal distribution, median and interquartile range (IQR), and for qualitative variables, frequency and percentages were calculated.

## Results

### Summary of demographic, clinical, immunological, and molecular features

Twelve Iranian patients with a clinical and molecular diagnosis of DCLRE1C deficiency were identified, three of whom were excluded due to the unavailability of adequate clinical information. A total number of nine patients with *DCLRE1C* mutation (6 males and 3 females) from nine families were enrolled in this study. All patients, except P4 and P7, had consanguineous parents. All patients were born full-term with normal birth history. Family history of immunodeficiency disorders or early death was recorded in none of the patients’ documents, except for P3 whose older brother had died due to recurrent pneumonia at the age of 3 years old. A history of spontaneous abortion was observed in the family of 33.3% of patients. The median (IQR) age of onset was 6.0 (5–17.0) months. Severe combined immunodeficiency (SCID) was clinically detected at a median (IQR) age of 7.0 (6–20.5) months, following a median (IQR) diagnostic delay of 2.0 (1.0–3.5) months (Table 1).

**Table 1:** Summary of demographic, and clinical findings in Artemis deficiency

ID	Alive/dead	Cause of death	Age at diagnosis(m)	Age at onset (m)	Diagnostic delay (m)	Age at death (m)	Sex	Failure to thrive	Consanguinity of parent	Recurrent infections in family member
P1	Dead	Pulmonary hemorrhage	3	2	1	12	M	Negative	Positive	Negative
P2	Dead	Cardiopulmonary arrest	7	6	3	7	F	Negative	Positive	Negative
P3	Alive	–	19	15	4	–	F	Positive	Positive	Positive (older brother's death due to pneumonia at 3 years old
P4	Dead	Pneumonia	6	6	0	48	F	Negative	Negative	Negative
P5	Dead	Encephalitis	132	120	12	156	M	Negative	Positive	Negative
P6	Dead		10	8	2	12	M	Negative	Positive	Negative
P7	Dead	Sepsis	6	5	1	10	M	Negative	Negative	Negative
P8	Dead	Cardiopulmonary arrest	6	5	1	18	M	Negative	Positive	Negative
P9	Alive	–	22	19	3	–	M	Positive	Positive	Negative

ID	History of Abortion	Immunodeficiency disorders in family	First clinical feature	First clinical diagnosis	Site of infection	Relapse after treatment	Autoimmune disease	Allergic disease	Malignancy
P1	Once	Negative	Fever, otorrhea, cradle cap, skin rashes, scaling skin	Bilateral otitis media	Both ears	Positive	Negative	Anaphylaxis due to contrast dye, cow's milk intolerance	Negative
P2	Negative	Negative	Left axillary lymphadenopathy and lymph node abscess after BCG vaccination	BCG lymphadenitis	Left axillary lymph node	Negative	Negative	Cow's milk intolerance	Negative
P3	Twice (induced abortion)	Negative	Ulcer and vesicular lesions in mouth, throat, anus with low-grade fever, diarrhea, otorrhea, mastoiditis, otitis media	Mastoiditis and otitis media	Right ear	Positive	Negative	Negative	Negative
P4	Negative	Negative	Cough, fever, diarrhea	Pneumonia	Lungs	Negative	Negative	Negative	Negative
P5	Once	Negative	Diarrhea. cough, fever, otitis media, arthralgia	Pneumonia, otitis media	Lungs and both ears	Positive	juvenile idiopathic arthritis	Negative	Negative
P6	Negative	Negative	Diarrhea, cough,	Pneumonia	Lungs	Positive	Negative	Negative	Negative
P7	Negative	Negative	Fever, cough, diarrhea, lymphadenopathy, hepatosplenomegaly	Pneumonia	Lung and GI	Negative	Negative	Negative	Negative
P8	Negative	Negative	Axillary lymph node abscess and splenomegaly	BCGosis	Left axillary lymph node	Negative	Negative	Negative	Negative
P9	Once	Negative	Severe Diarrhea and FTT	Gastroenteritis	Intestine		ITP, Celiac disease	Negative	Negative

BCG, Bacilli Calmette-Guerin; FTT, failure to thrive; F, female; GI, gastrointestinal; ITP, immune thrombocytopenic purpura; M, male

The most common initial presentation was pneumonia (44.4%) and otitis media (33.3%), followed by Bacillus Calmette–Guérin (BCG) lymphadenitis (22.2%) and gastroenteritis (11.1%). The most prevalent manifestations during entire course of the disease were respiratory tract infections (including otitis media) (66.6%) and chronic diarrhea (66.6%). Three patients developed lymphoproliferation (two axillary lymphadenopathies following BCG vaccination, two splenomegaly, and one hepatomegaly). Two patients suffered from mucocutaneous lesions and failure to thrive (FTT). Moreover, autoimmunity was found in two patients, one patient had juvenile idiopathic arthritis (P5) and the other had celiac disease and idiopathic thrombocytopenic purpura (ITP) (P9). Two patients (P1 and P2) presented with allergic disorders in the form of food allergy and anaphylaxis. Malignancy was not found in any of the patients during median (IQR) 12 [3–33] months follow-up period.

In the immunologic evaluation, all patients were lymphopenic, except P1, P5 and P9 who had normal lymphocyte count. A lymphocyte subset study using flow cytometry revealed normal NK cell count, and reduced B cell and T CD4+ cell counts in all patients. In addition, T CD3+ and CD8+ cell counts were reduced in 7 patients (77.8%). Six patients (66.6%) were found to have low IgG serum levels. Low serum levels of IgA, IgM and IgE were detected in 77.8%, 55.5%, and 22.2% of individuals, respectively. Specific antibody response to diphtheria and tetanus was measured in 3 patients and was absent in all of them (Table 2). Genetic analysis was performed for 9 patients. Homozygous *DCLRE1C* mutation was found in all cases. Two patients from unrelated families had the same homozygous mutation in *DCLRE1C* (c.632G > T, p.G211V).Further details on mutation and related clinical features are discussed in the detailed information on the study population. All patients received Ig replacement therapy and prophylactic antibiotics. P1 was the only patient who underwent HSCT from his HLA-matched grandmother at the age of 11 months. Eventually, 11 days after transplantation died due to respiratory distress and pulmonary hemorrhage. For two patients, who are alive at the time of the current report, the severity and number of infections decreased. Unfortunately, other patients died due to disease-related complications.

**Table 2:** Summary of Immunologic and Genetic data in Artemis disease

ID	WBC (×10^3^ /µl)	Lymphocyte (cell/µl)	Plt (cell*10^3^/µl)	Hb (g/dl)	CD3 (cell/µl)	CD4 (cell/µl)	CD8 (cell/µl)	CD19 (cell/µl)	NK (cell/µl)	IgG (mg/dl)	IgA (mg/dl)	IgM (mg/dl)	IgE (IU/ml)	Anti-tetanus toxin IgG (mIU/ml)	Anti-Diphtheria toxin IgG (mIU /ml)	Mutation in *DCLRE1C*
P1	11.0 NL	3740 (34%) NL	685 H	10.5 NL	250.5 (6.7%) L	235.62 (6.3%) L	11.2 (0.3%) L	22.4 (0.6%) L	1555.8 (41.6%) NL	80 L	< 7 L	< 7 L	0.1 NL	ND	ND	Hom large deletion of exons 1–4
P2	14.3 NL	2860 (20%) L	439 NL	10.3 L	< 1 L	< 1 L	< 1 L	< 1 L	1601.6 (56%) NL	835 NL	8 L	6 L	0.1 NL	0.02 L	0.001 L	Hom c.362 + 1G > T
P3	4.5 L	1255.5 (27.9%) L	380 NL	9.6 L	615.1 (49%) L	75.3 (6%) L	301.3 (24%) L	6.2 (0.5%) L	338.8 (27%) NL	446 NL	5 L	134 NL	3 NL	ND	ND	Hom c.632G > T, p.G211V
P4	5.5 L	3405 (68.1%) L	411 NL	12 NL	2213 (65%) NL	544 (16%) L	1634.4 (48%) NL	68.1 (2%) L	1770.6 (54%) NL	52 L	Undetectable	33 L	0.1 NL	ND	ND	Hom c.632G > T, p.G211V
P5	4.4 L	2217 (50.4%) NL	244 NL	13.3 NL	257 (11.6%) L	168 (7.59%) L	101 (4.58%) L	Undetectable	1773 (80%) NL	173 L	3 L	11 L	2 NL	< 0.1 L	< 0.1 L	Hom c.1250_1260 delCAATGACTGCA, p.S417YfsX459
P6	3.4 L	2040 (60%) L	460 H	10.7 NL	748 (22%) L	102 (5%) L	306 (15%) L	61 (3%) L	958, (47%) NL	90 L	15 NL	55 NL	15 L	ND	ND	Hom c. 1162 G > A, p.E388X
P7	8.2 NL	336.2 (4.1%) L	200 NL	11 NL	14.7 (4.4%) L	4.3 (1.3%) L	6.7 (2%) L	5.7 (1.7%) L	171.4, (51%) NL	371 NL	23 NL	82 NL	89 H	ND	ND	Hom c.329 T > G, p.L110R
P8	4.4 NL	442 (10%) L	671 H	9 L	1.7, (0.4%) L	19.89 (4.5%) L	30.9 (7%) L	4.4 (1%) L	397 (90%) NL	15 L	6.2 L	Undetectable	23 H	ND	ND	Hom large deletion of exons 1–3
P9	9.5 NL	2470 (26%) NL	555 H	10.2 NL	1457 (59%) NL	422 (17.1%) L	1037.4 (42%) NL	197.6 (8%) L	1432.6 (58%) NL	380 L	< 5 L	48 NL	ND	0.01 L	0.03 L	Hom c.41G > T, p.G14V

H, high Hb, hemoglobin; Ig, immunoglobulin; L, low; ND, not done; NL, normal; NK, natural killer cell; Plt, platelet; WBC, whole blood cells

### Detailed information of the study population

P1 was the second child of consanguineous parents. His family history was unremarkable and his older brother was healthy. He was born full-term and had a normal birth history. Medical history included recurrent otitis media and skin rashes since 40 days of age with relapse after therapies with oral antibiotics. A history of food allergy was reported (the type of food was not mentioned) and the patient had an anaphylactic reaction in the form of dyspnea and flushing after a CT scan with contrast. At 2 months of age, he was admitted to the hospital with fever and otorrhea associated with cradle cap, diffuse skin rashes, and scaling skin rash. He received IV antibiotics (ceftriaxone 50 mg/kg) and corticosteroids (oral prednisolone and IV hydrocortisone). Further investigations confirmed generalized lymphadenopathy with some areas of central necrosis. Laboratory findings showed normal total lymphocyte count and normal NK cells, while reduced T CD3^+^, T CD4^+^, T CD8^+^ and B CD19^+^ cell count. Serum levels of immunoglobulins (IgG, IgM, IgA) were low. The lymphocyte transformation test (LTT) in response to BCG (1.2, normal ≥ 2.5) and phytohemagglutinin (PHA) stimulation was impaired (2.5, normal range ≥ 3). HIV real-time reverse transcription–polymerase chain reaction (RT-PCR) was negative. Genetic analysis revealed the deletion of exons 1 to 4 in the *DCLRE1C* gene. At 4 months of age Ig replacement therapy and prophylaxis with co-trimoxazole, acyclovir, fluconazole and isoniazid were started. He underwent HSCT from his HLA-matched grandmother at 11 months of age. However, 11 days after transplantation presented with pancytopenia and reduced systemic oxygen saturation, and finally died due to respiratory distress and pulmonary hemorrhage.

P2 was the only child of consanguineous parents. She was born full-term with normal birth and family history. Cow’s milk intolerance was reported. At 6 months of age, she presented with peripheral lymphadenopathy (left axillary and supraclavicular), abscess formation in the left axilla, and purulent discharge associated with swelling and erythema at the site of BCG vaccine injection. The blood culture was positive for Pseudomonas aeroginosa. Laboratory findings demonstrated low lymphocyte count and reduced all main lymphocyte subsets. Specific antibodies for tetanus and diphtheria were unprotective. The normal level of serum IgG associated with low IgA and IgM levels was detected. Nitroblue tetrazolium (NBT) was normal. Genetic analysis revealed a homozygous splicing mutation in the *DCLRE1C* gene (c.362 + 1G > T). She underwent axillary lymph node dissection, abscess drainage, and received Ig replacement therapy, anti-tuberclosis treatment with rifampin, ethambutol and isoniazid accompanied by IV vancomycin and meropenem. The specimen of left axillary abscess was positive for numerous acid fast bacili. She was discharged from the hospital with infection prophylaxis (metronidazole, cephalexin, itraconazole). After two weeks she was admitted to the hospital with multiple abscesses and lymphadenitis (axillary and supraclavicular), respiratory distress and pancytopenia. Eventually, she died due to septicemia at age 7 months.

P3 was the third child of consanguineous parents. She was born full-term with a normal birth history. Her older brother had died due to recurrent pneumonia when he was 3 years old, however, he was not assessed for immune deficiency.The second child was healthy. At 19 months of age, she presented to the hospital with mild diarrhea, low-grade fever, FTT, and cutaneous rashes around the mouth, genitalia, buttock, and both legs. Her medical history included recurrent skin rashes and mastoidectomy due to otitis media complications at 14 months of age. Further laboratory findings demonstrated low lymphocyte count and reduced all lymphocyte cell subsets but normal NK cell count. Moreover, the cytomegalovirus (CMV) PCR test in the whole blood sample was positive. Serum IgA was low, while serum IgG, IgM, and IgE were normal. The NBT test was normal and the LTT revealed a normal response to PHA (3.5, normal range ≥ 3), but it was impaired in response to BCG (1.7, normal range ≥ 2.5) and candida (1, normal range ≥ 2.5) stimulation. Genetic analysis revealed a homozygous missense mutation in *DCLRE1C* (c.632G > T, p.G211V). Monthly Ig replacement therapy and prophylaxis with co-trimoxazole, itraconazole and acyclovir were started. She is now 5 years old. She responded well to therapy, and a reduction in the rate and severity of infection was noted and now is in the waiting list for HSCT.

P4 was the only child of nonconsanguineous parents. She was born full-term with a normal birth history. At 6 months of age, she presented to the hospital with cough and diarrhea, unresponsive to outpatient treatment. Laboratory findings demonstrated low lymphocyte count, normal T CD3+ and T CD8+ accompanied by low T CD4^+^ and B CD19^+^ cell counts, and normal NK cell count. Serum IgG, IgM, and IgA were diminished. While the NBT test was normal. CMV DNA was detectable in the whole blood sample. Genetic analysis revealed the same homozygous mutation in the *DCLRE1C* gene (c.632G > T, p.G211V). At the age of 7 months, Ig replacement therapy was commenced. Finally, at the age of 4 years, she died due to severe pneumonia.

P5 was the second child of consanguineous parents. He was born full-term with normal birth history. The first child was spontaneously aborted. At the age of 10 years, he presented with fever, cough, and arthralgia. Medical history included oral candidiasis at two years, recurrent otitis media, skin rashes, juvenile idiopathic arthritis and severe osteoporosis. Laboratory findings demonstrated normal NBT test, normal total lymphocyte and expanded NK cell count, while decreased T and B cell counts. Low levels of IgG, IgM, and IgA were detected. Specific antibodies for tetanus and diphtheria were unprotective. Genetic analysis revealed homozygous frameshift *DCLRE1C* mutation (c.1250_1260delCAATGACCTGCA, p.S417YfsX459) and Ig replacement therapy was commenced. Unfortunately, he died due to encephalitis at 13 years of age.

P6 was the second child of consanguineous parents. He was born full-term with normal birth history. No information about the health status of the first child has been reported. At 8 months of age, he was admitted to the hospital with cough, diarrhea, and respiratory distress. His medical history included recurrent respiratory infections. Laboratory findings demonstrated low lymphocyte count, reduced T CD3^+^, T CD4^+^, T CD8^+^ and B CD19^+^ cells count, but normal NK cells count. Serum IgG level was low, whereas serum IgM and IgA were within normal range. Genetic analysis revealed a homozygous stopgain mutation in the *DCLRE1C* gene (c.1162G > A, p.E388X). Although he received infection prophylaxis with co-trimoxazole, itraconazole and acyclovir, Finally, he died of severe pneumonia at the age of 12 months.

P7 was the only child of nonconsanguineous parents. He was full term with normal birth history. Medical history and family history were unremarkable. At 5 months of age, he presented to the hospital with fever, cough, diarrhea, generalized lymphadenopathy, and hepatosplenomegaly. Laboratory findings demonstrated low lymphocyte count, reduced T CD3^+^, T CD4^+^, T CD8^+^, B CD19^+^ cell counts, and normal NK cell count. Serum immunoglobin production including IgG, IgA, and IgM were all normal. Genetic analysis revealed a homozygous missense mutation in the *DCLRE1C* gene (c.329 T > G, p.L110R). Finally, at the age of 7 months, he died due to sepsis.

P8 was the third child of consanguineous parents. He was born full-term with normal birth history and family history. Other sibilings were healthy. At the age of 5 months he was admitted to the hospital with several complaints including fever, oral candidiasis, erythema and swelling at the site of the BCG vaccination, disseminated lymphadenopathy, and hepatomegaly. The BCGosis was confirmed and he underwent anti-tuberculosis treatment with rifampin, isoniazid, and ethambutol, Further laboratory findings demonstrated low lymphocyte count, reduced all lymphocyte subsets including CD3, CD4 and CD8 T cell and CD19 B cell, despite normal NK cells count. Serum immunoglobulin (IgG, IgM, IgA) levels were low. Genetic analysis revealed a homozygous large deletion (Exon 1-3) in the *DCLRE1C* gene. Immunoglobin replacement and prophylaxis with co-trimoxazole, acyclovir and fluconazole were commenced. Unfortunately, at the age of 10 months, he died due to cardiopulmonary arrest.

P9 was the second child of consanguineous parents. He was born full-term with normal birth history. The first child was spontaneously aborted. At 19 months of age, he presented with FTT and diarrhea. His medical history included recurrent respiratory infection and celiac disease since he was 5 months. Additionally, at 2 years old, diagnosis of ITP was confirmed. Further laboratory findings demonstrated normal lymphocyte count, normal T CD3^+^, T CD8^+^, NK cells count, and reduced T CD4^+^ and B CD19^+^ cells count. Serum IgG and IgA levels were low but IgM was normal. Genetic analysis revealed a homozygous missense mutation in the *DCLRE1C* gene (c.41G > T, p.G14V). He is now 24 years old. He underwent Ig replacement therapy. He responded well and a reduction in the rate and severity of infections was noted.

## Discussion and conclusion

To prevent delay in the diagnosis of future Artemis cases and expand the knowledge regarding clinical, laboratory and demographic characteristics of Artemis deficiency we performed the current nationwide study. Mutation in *DCLRE1C* in Iran was found in 0.5% of patients with genetically diagnosed SCID [12]. While the prevalence of T-B-NK+ in the United States has been estimated approximately1:60,000 live births [16]. Most of the patients in this study had consanguineous parents (77.7%). Consanguineous marriage has been reported as a contributing factor in other studies [7, 17, 18]. In a report of 14 patients from 10 families who had combined immunodeficiency due to the Artemis mutation,4 families were consanguineous [17]. According to the relatively high rate of consanguine marriage in Iran, more patients with SCID phenotype are expected to be identified, in a study by Aghamohammadi et al. in 2021, 17,120 patients with IEIs from 22 countries in the Middle East and North Africa (MENA) were identified and 60.5% of cases were born in the consanguineous family [15]. The high prevalence of consanguineous marriage 20–50% in MENA countries can explain a large number of patients with IEIs in these countries [15, 19, 20]. Jamee et al. estimated the rate of consaguinity mariage about 76% in MENA countries [20]. but due to the lack of proper screening programs, a notable proportion of patients die before confirmed diagnosis. Furthermore, there is a need for more accurate and comprehensive demographic, clinical and immunological data from the patients for further investigations in the future.

The median diagnostic delay was estimated at 2.0 months. There has been a reduction in the combined T- and B-cell immunodeficiencies diagnosis delay in Iran from the year 2006 [18], whereas it has remained unchanged since 2019 [8]. The decrease in diagnosis lag since 2006 may reflect the recent rise in knowledge and understanding of immune deficiency. Moreover, in a study by Abolhassani et al. in 2018, the median diagnosis delay among PID patients in Iran was reported 10 months. Although, in a five year-study only a slight difference was observed in diagnosis delay of new patients versus old registered cases [12]. In the report from IEIs patients in MENA countries, the median diagnosis delay of IEI was predicted about 41 months [15]. In 2010, SCID was included in the newborn screening (NBS). Currently, most states in the United States and some of the European countries carry out a newborn screening program for SCID utilizing an analysis to identify T-cell receptor excision circles (TREC) in dried blood spots [21, 22]. Although, recent advances in newborn screening have enabled an early detection of IEIs in asymptomatic infants, they are not universally available. In addition, some patients with hypomorphic SCID may escape the TREC screening test and there may be a need for the addition of targeted sequencing as a second-tier test in the future [23, 24]. Therefore, currently most patients with chromosomal instability syndromes but less severe symptoms are misdiagnosed or exposed to frequent radioactive imaging following respiratory tract complications which increases their chance of developing malignancy and death before the establishment of a definite diagnosis [15]. CXR or CT imaging may raise the risk of cancer in Artemis patients due to radiosensitivity and DNA repair deficiencies. Therefore, a preliminary assessment based on clinical features and pulmunary function test (PFT) patterns prior to imaging is advised [25, 26]. The diversity in the clinical and laboratory features of Artemis deficiency may challenge the early diagnosis of patients and lead to irreversible life-threatening sequelae.

Induced abortion was observed in 33.3% of cases. Based on previous researches poor pregnancy outcomes may be attributed to immunological aberrancy. Recurrent spontaneus abortion may result from the complex interactions between different immune cells, altering the stability between immunological response and empryonic antigen tolerance through a variety of mechanisms [27].

Respiratory infections were the most common clinical manifestation, although the intensity of the symptoms varied among individuals. In a report by.Lee et al. in 2013 recurrent respiratory infections and candidiasis were the most frequent manifestations [17]. Consistent with this report and previous investigations in Iran [18]. In this study, juvenile idiopathic arthritis affected P5, celiac disease and idiopathic thrombocytopenia affected p9, while malignancy was not reported. The spectrum of manifestations may vary according to geographical regions. In a report from Israel, for instance, the majority of the patients primarily presented with autoimmune features [7]. In a cohort analysis of 15 individuals with the *DCLREIC* mutation by Felgentreff et al., autoimmune cytopenia was found in one patient while other autoimmune manifestations and malignancy were detected in 2 and 5 patients, respectively [28]. In another large cohort research by Lankester et al., autoimmune manifestation was discovered in one out of 34 patients [29] (Additional file 1: Table S1). In addition, the degree of Artemis residual function due to the hypomorphic mutation as well as functional innate immunity is the determining aspects that can affect the severity and prognosis of the disease. In this investigation growth retardation was seen in 12 patients (22.2%), compared to 5 patients (14.7%) in an earlier cohort study [29]. Further details comparing clinical features and genetic mutations are summeraized in Additional file 1: Table S1.

Patients underwent a basic immunological examination quite early in their lives and the most notable result was a reduction in B cells in all patients as well as a reduced concentration of IgG level (66.6%) and T cell count in the majority (77.8%) of individuals. Additionally, IgA and IgM deficiency was detected in 77.8% and 55.5% of patients respectively. These outcomes are in agreement with an earlier study, as Sundin et al. 2019 reported a 3-year-old girl with DCLRE1C gene mutation and undetectable immunoglobulins (IgG, IgA, IgM) associated with diminished CD3 and CD19 cells [3]. Additionally in a study by Volk et al. laboratory data from 3 patients with DCLRE1C mutation revealed low B cell and IgA levels in combination with low naïve T cells [30]. Whereas, a survey by Nahum et al. presented 4 patients with DCLRE1C hypomorphic mutation, contrary to most studies IgG hypergammaglobulinemia along with IgA and IgE deficiency was observed. It is assumed that IgA and IgE deficiency is due to the location of the genes which is on the same chromosome next to another and the absence of typical class switch recombination [7].

Typical SCID without effective therapy is always deadly in infancy. To have a chance of survival, patients need timely management and proper therapy. For patients with T-B- SCID, HSCT is the only curable treatment, although, the outcomes vary depending on the type of donor available and the conditioning utilized [14, 31]. Moreover, the recent advancement in gene therapy has proposed promising alternative treatment for SCID. Compared to other types of SCID, Artemis Patients report more severe conditioning-related side effects [32]. Those who receive HSCT from matched sibiling donors survive at an 85% rate, whereas patients who receive it from haplo-identical donors survive at a 65% rate [11]. A clinical trial used DCLRE1C expressing lentiviral vector transduced CD34+ hematopoetic stem cell and 3 of 3 cases showed normal mitogene lymphocyte proliferation.The only side effect was autoimmune hemolytic anemia, which occurred in two patients [33]. In our study, P1 received HSCT at 11 months of age from an HLA-matched related donor and died of respiratory hemorrhage 11 days after transplantation. Patients living in developing countries face several challenges regarding bone marrow transplantation, including a lack of immunologists with expertise in HSCT, long waiting list for finding compatible donor, and limited practice of haploidentical transplant. In the report by Lankester et al., HSCT 2-year overal survival in DCLRE1C-deficient SCID was estimated about 80% [29]. A study from the UK assessed long-term outcomes and immunological recovery in Artemis deficiency post-HSCT. Artemis patients showed more persistent complications such as dental and dermatological problems accompanied by short stature in comparison to IL7Rα and RAG1/2 SCID. In addition, when comparing conditioned and unconditioned recipients, more CD4^+^ naïve lymphocytes were seen post-HSCT in conditioned recipients [34]. Another investigation revealed Artemis and RAG deficient do not differ in survival after HSCT. However, the use of alkylating drugs before HSCT is significantly correlated with poor growth in Artemis deficient patients [11]. The classic SCID patients who do not undergo HSCT can not survive beyond infancy, nonetheless, those with hypomorphic features may survive by conventional therapy. Two of our patients are currently alive (5 years old and 24 years old) and the frequency and severity of infections have decreased by receiving IVIG. Regarding the variations in disease severity and progression among patients who carry the same mutation, we can only speculate that this difference may be caused by hypomorphic mutations, various genetic backgrounds, unidentified disease-modifying genes or exposure to various pathogens at different phases of the disease’s progression [7, 17]. The findings of the this study mostly confirm the findings of previous reports, however, it has several limitations including a retrospective nature, limited number of participants, and incomplete data regarding autoantibody screening, vaccination titer, and infecting microorganisms. Therefore, further prospective studies with greater study population and natural history studies for better characteriziation of Artemis deficiency phenotype are recommended to be undertaken.

Early detection of SCID is important to prevent the development of life-threatening infections and to allow for rapid curative stem-cell transplantation. It must be taken into account that recurrent infections during the first months of life are a prominent presentation of immunodeficiency disorders, even in the presence of normal growth and development.

## Supplementary Information

**Additional file 1: Table S1.** Comparison of the current cohort of DCLRE1C deficiency with previously large cohort of patients.

## Data Availability

Patients’ data are available via reseanoble request to corresponding authors.

## Abbreviations

RS-SCID: Radiosensitive severe combined immunodeficiency
SCID: Severe combined immunodeficiency
ITP: Immune thrombocytopaenia purpura
NHEJ: Non-homologous end-joining
HSCT: Hematopoietic stem cell transplantation
IPIDR: Iranian National Registry of Primary Immunodeficiencies
LTT: Lymphocyte transformation test
FTT: Failure to thrive
